# Development of real-time and lateral flow dipstick recombinase polymerase amplification assays for rapid detection of goatpox virus and sheeppox virus

**DOI:** 10.1186/s12985-017-0792-7

**Published:** 2017-07-17

**Authors:** Yang Yang, Xiaodong Qin, Xiangle Zhang, Zhixun Zhao, Wei Zhang, Xueliang Zhu, Guozheng Cong, Yanmin Li, Zhidong Zhang

**Affiliations:** State Key Laboratory of Veterinary Etiological Biology, Lanzhou Veterinary Research Institute, Chinese Academy of Agriculture Sciences, Xujiaping 1, Lanzhou, Gansu 730046 China

**Keywords:** Recombinase polymerase amplification, CaPV real-time RPA, CaPV RPA LFD, Goatpox virus, Sheeppox virus

## Abstract

**Background:**

Goatpox virus (GTPV) and sheeppox virus (SPPV), which belong to the *Capripoxvirus* (CaPV), are economically important pathogens of small ruminants. Therefore, a sensitive, specific and rapid diagnostic assay for detection of GTPV and SPPV is necessary to accurately and promptly control these diseases.

**Methods:**

Recombinase polymerase amplification (RPA) assays combined with a real-time fluorescent detection (real-time RPA assay) and lateral flow dipstick (RPA LFD assay) were developed targeting the CaPV G-protein-coupled chemokine receptor (GPCR) gene, respectively.

**Results:**

The sensitivity of both CaPV real-time RPA assay and CaPV RPA LFD assay were 3 × 10^2^ copies per reaction within 20 min at 38 °C. Both assays were highly specific for CaPV, with no cross-reactions with peste des petits ruminants virus, foot-and-mouth disease virus and Orf virus. The evaluation of the performance of these two assays with clinical sample (*n* = 107) showed that the CaPV real-time RPA assay and CaPV RPA LFD assay were able to specially detect SPPV or GTPV present in samples of ovine in liver, lung, kidney, spleen, skin and blood.

**Conclusions:**

This study provided a highly time-efficient and simple alternative for rapid detection of GTPV and SPPV.

**Electronic supplementary material:**

The online version of this article (doi:10.1186/s12985-017-0792-7) contains supplementary material, which is available to authorized users.

## Background

Sheeppox virus (SPPV) and goatpox virus (GTPV) belong to the genus *Capripoxvirus* (CaPV), subfamily *Chordopoxvirinae*, family *Poxviridae*, and cause serious pox diseases of domesticated small ruminants [1, 2]. Sheeppox and goatpox are endemic in most of Asia, the Middle East and North Africa and classified as notifiable diseases by World Organization for Animal Health (OIE, 2013, accessed 10.23.13) [3, 4]. For accurately and promptly controlling any outbreak, the foremost requirement is the sensitive, specific and rapid tool for detection of the causative agents [5, 6]. Although various methods such as virus isolation, serology tests and polymerase chain reaction (PCR) [7–10] are available for diagnosis of these diseases, these tests have certain limitations such as time-consuming, laborious and technical complexity [6, 11, 12]. PCR based-diagnostic assay relies on expensive instrumentation such as thermal cycler and usually takes over more than one hour to complete, which make it hard to be used in poorly equipped laboratories or as a pen-site test in the field.

Recombinase polymerase amplification (RPA) is a novel isothermal alternative to PCR, which can amplify detectable amount of DNA in 20 min or less with simple instrumentation. RPA employs recombinases to anneal oligonucleotide primers to template DNA for extension and amplification by a polymerase at an isothermal temperature [13]. Real-time detection of RPA amplicons could be performed through TwistAmp exo probes (TwistDx, Cambridge, UK). Fluorescence accumulation relies on the separation of fluorophore and quencher through exonuclease III. As an alternative to real-time detection, RPA amplicons could also be visualized on lateral flow dipstick with TwistAmp nfo probe. Currently, RPA assay has been successfully developed for rapid detection of different viruses of veterinary importance, including foot-and-mouth disease virus [13], avian influenza H5N1 [14], Orf virus [15, 16], bovine viral diarrhea virus [17] and canine parvovirus type 2 [18]. The purpose of this study was to develop a RPA assay based on either real-time fluorescent detection (real-time RPA assay) or lateral flow dipstick (RPA LFD assay) for rapid detection of GTPV and SPPV and evaluate its performance in clinical samples.

## Methods

### Virus strains, reference DNA, and samples preparing and extracting

Goatpox virus (GTPV) /AV40, GTPV/AV41, GTPV/GS-V1, sheeppox virus (SPPV) /Gulang2009, SPPV/Jingtai2011, SPPV/Hubei, Orf virus (ORFV) /Vaccine/CHA, ORFV/HB/CHA, peste des petits ruminants virus (PPRV)/Nigeria 75/1, foot-and-mouth disease virus (FMDV)/O/CHA and FMDV/A/CHA were preserved in our laboratory. To prepare reference DNA, 374 bp CaPV GPCR segments (ranging from 301 bp to 675 bp of KF661979.1) were synthesized by Genewiz (Suzhou, China) and cloned into a pUC57 vector, designated as pCaPV/RPA. The pCaPV/RPA DNA was extracted by Plasmid Mini kit I (Promega, USA) and then measured by Nanovue (GE life science). The DNA copy number was calculated using the following equation: DNA copy number = (ng × 6.02 × 10^23^ × 10^−9^)/ (Fragment length (bp) × 660). The DNA standard was then aliquoted and stored at −80 °C until used. To prepare CaPV-spiked tissue lysates, CaPV-free tissue samples of liver, lung, stomach, kidney, lymphatic node, spleen, nasal swab and skin (*n* = 24, three each sample type) were collected from three healthy sheep. 10% (w/vol) tissue suspensions (one milligram tissue samples in nine volumes PBS) were then prepared by homogenizing tissue samples in PBS using MP FastPrep-24. Following a brief centrifugation, the homogenized tissue samples were spiked with SPPV/Gulang 2009 at different concentrations from 10^7^ copies to 10^4^ copies per reaction and stored at −80 °C until used. During the period of October 2014 to August 2015, one hundred and seven clinical samples (liver, lung, kidney, spleen, skin and blood) were collected from fourteen suspected sheep and six suspected goats in Gansu province which were characterized by pyrexia, excessive salivation and generalized pock lesions in the skin. Tissue samples were collected by the animal disease investigating teams based on good animal practices of the Animal Ethics Procedures and Guidelines of the People’s Republic of China (AEPGPRC), and the samples were stored at −80 °C in our laboratory until used. Nucleic acids from virus strains, spiked samples and clinical samples were extracted using the viral DNA/RNA extraction kit (TaKaRa, Dalian, China), which could only be used in the laboratory. In order to employ an energy-free assay in the field, a single innuPREP MP basic kit A (Jena Analytik, Jena, Germany) with a magnetic bead separation rack was tested using spiked samples (*n* = 24), and the extraction efficiency were then evaluated by real-time RPA assay, CaPV RPA LFD assay and CaPV real-time qPCR assay, respectively.

### Real-time qPCR assay

The real-time qPCR assay which could detect SPPV, GTPV and lumpy skin disease virus (LSDV) was carried out in an Agilent Technologies Stratagene Mx3005P thermocycler (Life technologies, USA) as previously reported [6]. Briefly, The PCR assay was carried out in a 25 μL reaction volume containing 2 × PCR buffer (a buffer containing 0.4 mM of each dNTP and 6 mM MgSO_4_, 12.5 μL), Taq DNA polymerase (5 U/μL, 0.5 μL), the probe (5`-CAATGGGTAAAAGATTTCTA-3`) (10 μM, 0.5 μL), the forward prime (5`-GGCGATGTCCATTCCCTG-3`) (10 μM, 1 μL), reverse primer (5`-AGCATTTCATTTCCGTGAGGA-3`) (10 μM, 1 μL), the DNA template (10 pg - 0.1 μg, 2 μL) and RNase-free water (9.5 μL). The cycling proceeded at 95 °C for 5 min, followed by 40 cycles of 95 °C for 50 s, 50 °C for 50 s and 72 °C for 1 min, and an additional extension for 5 min.

### RPA oligonucleotides and conditions

After blasting GPCR gene of CaPV (number of access: KF661979.1 (SPPV), KF661976.1 (SPPV), JQ310666.1 (SPPV), FJ869364.1 (GTPV), FJ869361.1 (GTPV), KP663705.1 (GTPV), KP719918.1 (LSDV), FJ869376.1 (LSDV) and KR024780.1 (LSDV)), one probe and three different forward and reverse primers targeting the GPCR gene conserved region were designed for each assay (CaPV real-time RPA and CaPV RPA LFD assay) and synthesized by Sangon Biotech (Table 1). The RPA assay was carried out in a 50 μL freeze-dried reaction tube, using 29.5 μL rehydration buffers (TwistDx, Cambridge, UK), 2.1 μL of each primer (10 μM), 0.6 μL probe (10 μM), 11.2 μL ultrapure water, 2 μL template and 2.5 μL magnesium acetate (280 mM). The real-time CaPV RPA assay was carried out using the TwistAmp exo kit (TwistDx, Cambridge, UK), and the fluorescence signal in the FAM channel (Excitation 470 nm, Detection 520 nm) was detected in an Agilent Technologies Mx3005P thermocycler for 60 cycles at 38 °C for 20 s. The RPA reaction was completed in 20 min. A sample was deemed positive if all replicates were three and a half standard deviations (3.5SD) above the background during a defined time range (i.e. after 19 to 20 min of amplification). A threshold time range of 0 to 4 min and 30 s was used. The CaPV RPA LFD assay was performed using the TwistAmp nfo kit (TwistDx, Cambridge, UK) at 38 °C for 20 min in a water bath. The LFD strips (Milenia Biotec GmbH, Germany) were used to detect amplified products. One μL of the amplified product was diluted in 99 μL of the assay buffer (Tris-buffered saline). The LFD strip were directly inserted into the mixture and incubated at an upright position for 2 min. A test was considered positive when the detection line and the control line were visible. A test was considered negative when only the control line was visible.

**Table 1:** Primers and probes used in CaPV real-time RPA and CaPV RPA LFD assay

Name	Sequence (5′ –3′)	Genome location (KF661979.1)
CaPV Fe1	CATTGTCTGATTTAATTTTCGTGTTGGTGTTTCCT	377–411
CaPV Fe2	CGTGTTGGTGTTTCCTTTTAATTTATACAATAGTA	396–430
CaPV Fe3	TGTTTCCTTTTAATTTATACAATAGTATAGCTAAA	404–438
CaPV Re1	ATCAATGTTATAAATGACATGCTATTGTAAAAACC	493–527
CaPV Re2	CAATAGCATGTCATTTATAACATTGATGAGTATTG	501–535
CaPV Re3	TATCTATCAATACTCATCAATGTTATAAATGACAT	508–546
CaPV Pe	TAAACAATGGAGTTTGGGAGATTGTTTGTG(FAM-	435–486
	dT)A(THF)A(BHQ1-dT)TCAAAGCTATGTTTTAC-P	
CaPV Fn1	CATTGTCTGATTTAATTTTCGTGTTGGTGTTTCCT	377–411
CaPV Fn2	CGTGTTGGTGTTTCCTTTTAATTTATACAATAGTA	396–430
CaPV Fn3	TGTTTCCTTTTAATTTATACAATAGTATAGCTAAA	404–438
CaPV Rn1	Biotin-ATCAATGTTATAAATGACATGCTATTGTAAAAACC	493–527
CaPV Rn2	Biotin-CAATAGCATGTCATTTATAACATTGATGAGTATTG	501–535
CaPV Rn3	Biotin-TATCTATCAATACTCATCAATGTTATAAATGACAT	508–546
CaPV Pn	FAM-AACAATGGAGTTTGGGAGATTGTTTGTGTA-THF-ATTCAAAGCTATGTTTTAC-P	435–486

“e” and “n” were defined as RPA exo kit and RPA nfo kit respectively. The expected size of amplification products for each pair of primers were as following: CaPV F1/CaPV R1 (151 bp), CaPV F1/CaPV R2 (159 bp), CaPV F1/CaPV R3 (170 bp), CaPV F2/CaPV R1 (132 bp), CaPV F2/CaPV R2 (140 bp), CaPV F2/CaPV R3 (151 bp), CaPV F3/CaPV R1 (124 bp), CaPV F3/CaPV R2 (132 bp) and CaPV F3/CaPV R3 (143 bp)

### Sensitivity and specificity of CaPV real-time RPA and CaPV RPA LFD assays

To determine sensitivity of CaPV real-time RPA assay and CaPV RPA LFD assay, the pCaPV/RPA DNA (ranging from 3 × 10^7^ to 3 × 10^1^ genome copies per reaction) was used in 8 replicates. RNase-free water was used as negative control reaction template in both CaPV real-time RPA assay and RPA LFD assay. The threshold time of CaPV real-time RPA assay was plotted against reference DNA molecules detected and the semi-log non-regression analysis was calculated with PRISM 5.0 software (GraphPad Software, USA). The probit analysis was performed using Statistica software (StatSoft, Hamburg, Germany). The specificity of both CaPV real-time RPA assay and RPA LFD assay was evaluated with nucleic acids extracted from different virus listed in Table 2. To determine the correlation of CaPV real-time RPA assay with CaPV real-time qPCR assay, both assays were tested with CaPV spiked samples (*n* = 24). The correction of CaPV real-time RPA assay threshold time (y axis) with CaPV real-time qPCR assay cycle threshold (CT) values (x axis) were generated by Excel software. To determine the optimum amplification temperature, CaPV RPA LFD assay was performed at a range of temperatures from 15 °C to 50 °C. The reaction time of CaPV RPA LFD assay was determined by terminating the RPA reaction at 0, 1, 5, 10, 15, 20, 25 and 30 min after the addition of magnesium acetate by immediate dilution and analysis on the lateral flow dipsticks.

**Table 2:** The specificity of CaPV real-time RPA assay and CaPV RPA LFD assay

Virus family	Virus specie	Virus strain	Real-time RPA	RPA LFD	Real-time qPCR
Poxviridae	Capripox	GTPV AV40	4.6 min	pos	19(CT)
		GTPV AV41	4.6 min	pos	20(CT)
		GTPV GS-V1	5 min	pos	20(CT)
	Capripox	SPPV Gulang2009	5.3 min	pos	21(CT)
		SPPV Jingtai2011	5 min	pos	21(CT)
		SPPV Hubei	5.3 min	pos	22(CT)
Poxviridae	ORFV	ORFV/Vaccine/CHA	neg	neg	neg
	ORFV	ORFV/HB/CHA	neg	neg	neg
Paramyxovirinae	PPRV	Nigeria 75/1	neg	neg	neg
Picornaviridae	FMDV	FMDV/O/CHA	neg	neg	neg
	FMDV	FMDV/A/CHA	neg	neg	neg

*pos* positive, *neg* negative, *GTPV* goat pox virus, *SPPV* Sheep pox virus, *ORFV* Orf virus, *PPRV* peste des petits ruminants virus, *FMDV* foot-and-mouth disease virus

## Results

### Sensitivity and specificity of CaPV real-time RPA assay

**Fig. 1:**
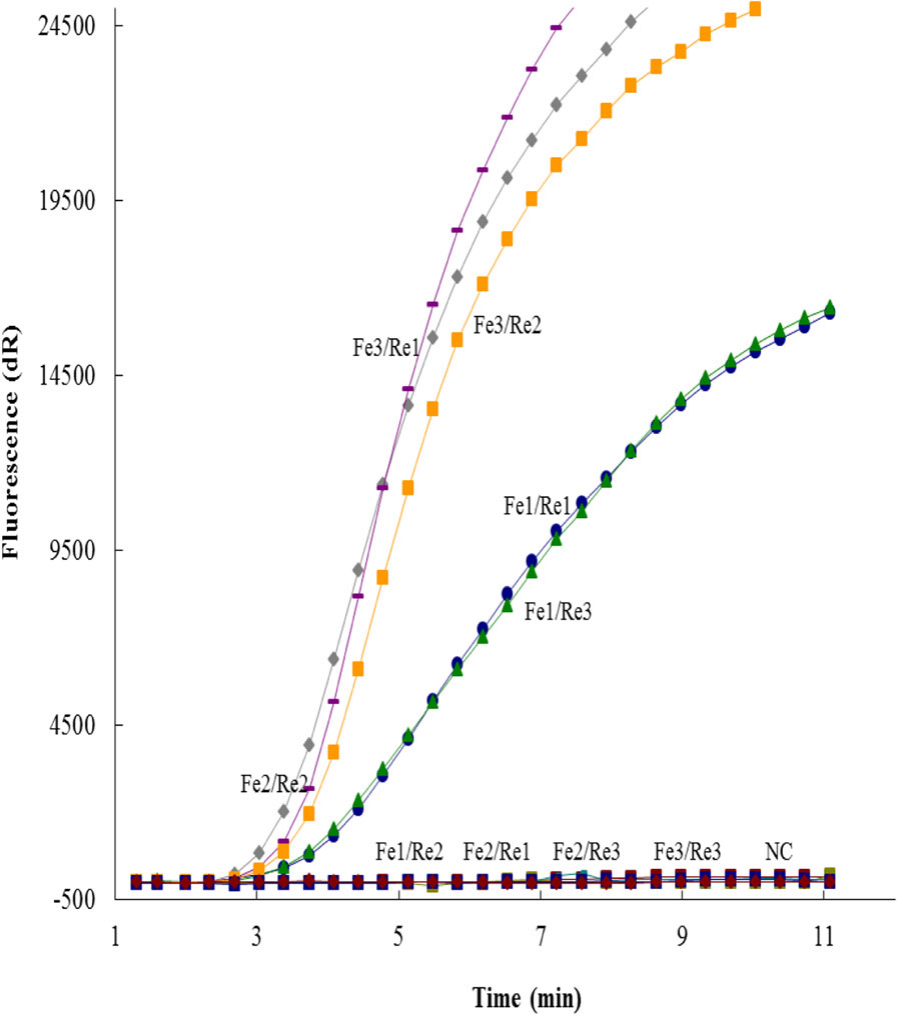
Optimal primers and probe combinations of CaPV real-time RPA assay. Three forward primers (CaPV Fe1 to CaPV Fe3), three reverse primers (CaPV Re1 to CaPV Re3) and one probe (CaPV Pe) were used to select the best combination. NC represents negative control

In initial optimization, nine combinations of candidate primers (3 forward and 3 reverse, Table 1) were generated and tested for the time to fluorescence threshold with the probe. Of these, five primer pairs produced signal with the probe, and three of the primer pairs (CaPV Fe3/CaPV Re1, CaPV Fe2/CaPV Re2 and CaPV Fe3/CaPV Re2) performed similarly and produced signal faster than other pairs (CaPV Fe1/CaPV Re1 and CaPV Fe1/CaPV Re3) (Fig. 1). Due to resource constraints, the primer pairs CaPV Fe2/CaPV Re2 were selected randomly for subsequent evaluation.

**Fig. 2:**
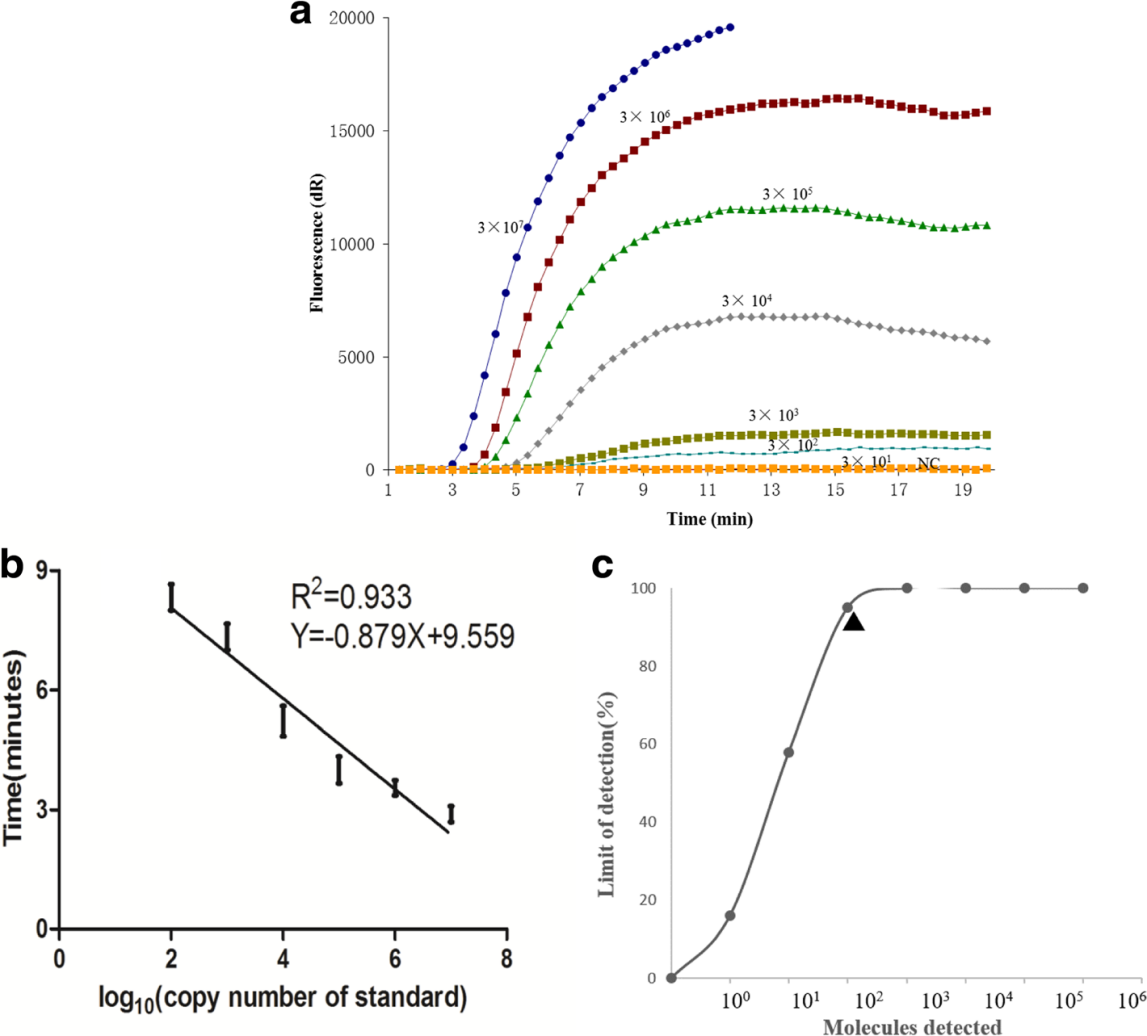
Sensitivity of real-time RPA assay (**a**) Typical raw fluorescence data of CaPV real-time RPA assay using a dilution series of the pCaPV/RPA DNA. NC represents negative control; (**b**) Reproducibility of the CaPV real-time RPA assay; (**c**) The limit of detection in 95% probability based on eight replicates

**Fig. 3:**
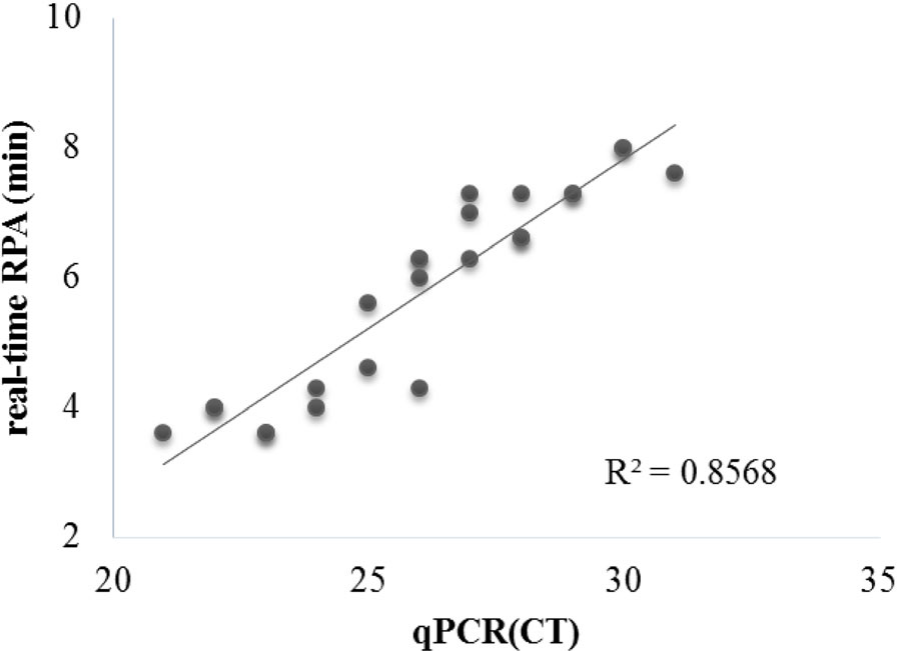
Comparison between performances of CaPV real-time RPA assay with real-time qPCR assay. The correction of CaPV real-time RPA assay threshold time (y axis) with CaPV real-time qPCR assay cycle threshold (CT) values (x axis) on CaPV spiked samples (*n* = 24) were generated by Excel software

To test the sensitivity of CaPV real-time RPA assay, serial dilutions of the purified reference DNA were tested for 8 replicates. As shown in Fig. 2, the dynamic detection range of the assay spans 5 logs ranging from 7 to 2 log copies per reaction, with the corresponding threshold time ranging from 3 min at 3 × 10^7^ copies per reaction to 7 min at 3 × 10^2^ copies per reaction at 38 °C. This result indicates that CaPV real-time RPA assay has a wide dynamic range for quantifying target DNA (Fig. 2a, b). The detection limit of CaPV real-time RPA assay at 95% probability was 3 × 10^2^ copies per reaction (probit analysis, *p* ≤ 0.05) (Fig. 2c). To further evaluate the sensitivity, the assay was tested with SPPV-spiked samples and compared with real-time qPCR assay. The results showed that both assays could detect viral DNA present in all the samples (Additional file 1: Table S1), and good correlation was found between threshold time (y axis) of CaPV real-time RPA assay and cycle threshold (CT) (x axis) of CaPV real-time qPCR assay (R squared 0.86, Fig. 3). In evaluation of the specificity of CaPV real-time RPA assay, consistent positive signal was only observed for GTPV strains (GTPV AV40, GTPV AV41, GTPV GS-V1) and SPPV strains (SPPV Gulang2009, SPPV Jingtai2011, SPPV Hubei), and no cross detection was observed with other viruses which can infect sheep and goats, including FMDV, ORFV and PPRV (Table 2). A simple DNA preparation method (single innuPREP MP basic kit with a magnetic bead separation rack) was also applied to spiked tissues samples. As shown in Table 3, CaPV real-time RPA assay performed well on these extracted DNA. There was no difference of the detection sensitivity between the two extraction methods when tested by CaPV real-time RPA assay (Table 3 and Additional file 1: Table S1).

**Table 3:** Extraction efficiency of the innuPREP MP basic kit on spiked samples (*n* = 24) were tested by real-time RPA assay, CaPV RPA LFD assay and CaPV real-time qPCR assay respectively

Sample name	Real-time qPCR (CT)	real-time RPA(min)	RPA LFD
liver 1	22	3.6	+
liver 2	27	4.3	+
liver 3	26	4	+
lung 1	31	7.6	+
lung 2	29	7.3	+
lung 3	24	4	+
stomach 1	26	4.3	+
stomach 2	28	4.3	+
stomach 3	32	8.3	+
kidney 1	22	4	+
kidney 2	25	4.6	+
kidney 3	29	7.3	+
lymphatic nodes 1	30	8	+
lymphatic nodes 2	21	3.3	+
lymphatic nodes 3	23	3.6	+
spleen 1	24	4.3	+
spleen 2	26	5	+
spleen 3	29	7.6	+
skin 1	22	4	+
skin 1	27	6.3	+
skin 1	28	6.6	+
nasal swab 1	25	6	+
nasal swab 2	26	6.3	+
nasal swab 3	28	7	+

### Sensitivity and specificity of CaPV RPA LFD assay

**Fig. 4:**
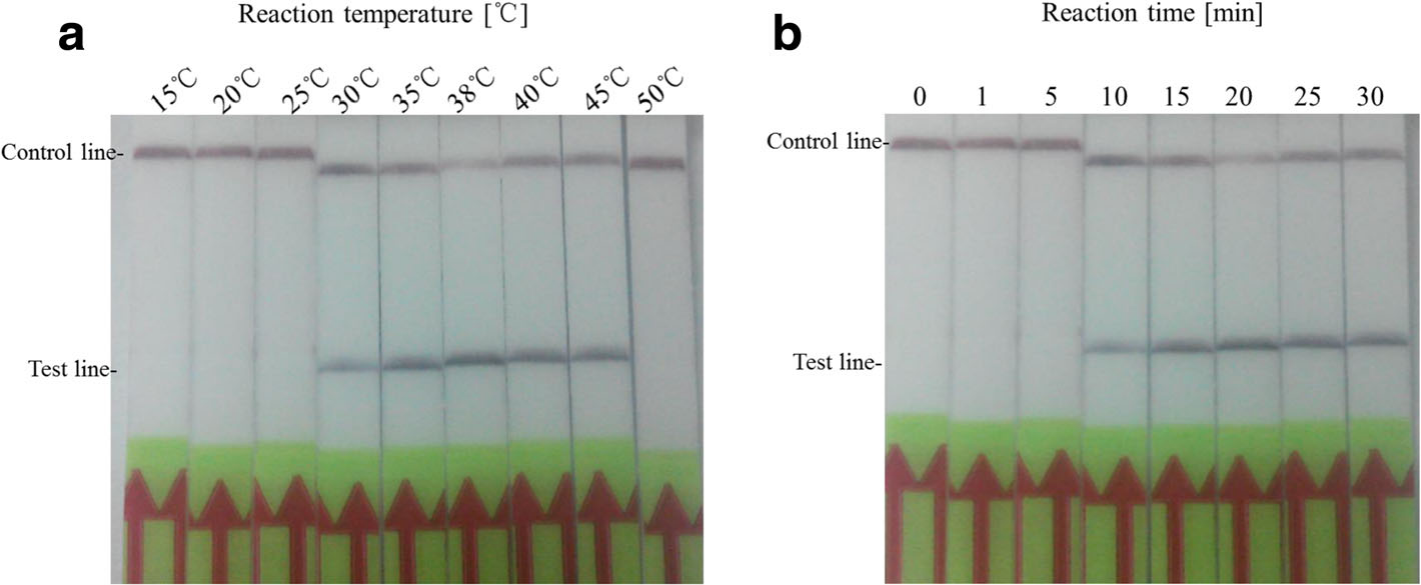
Determination of reaction temperature and time (**a**) CaPV RPA LFD assay are performed at different temperatures as shown. **b** The test line is visible at 38 °C when the amplification time is longer than 10 min

**Fig. 5:**
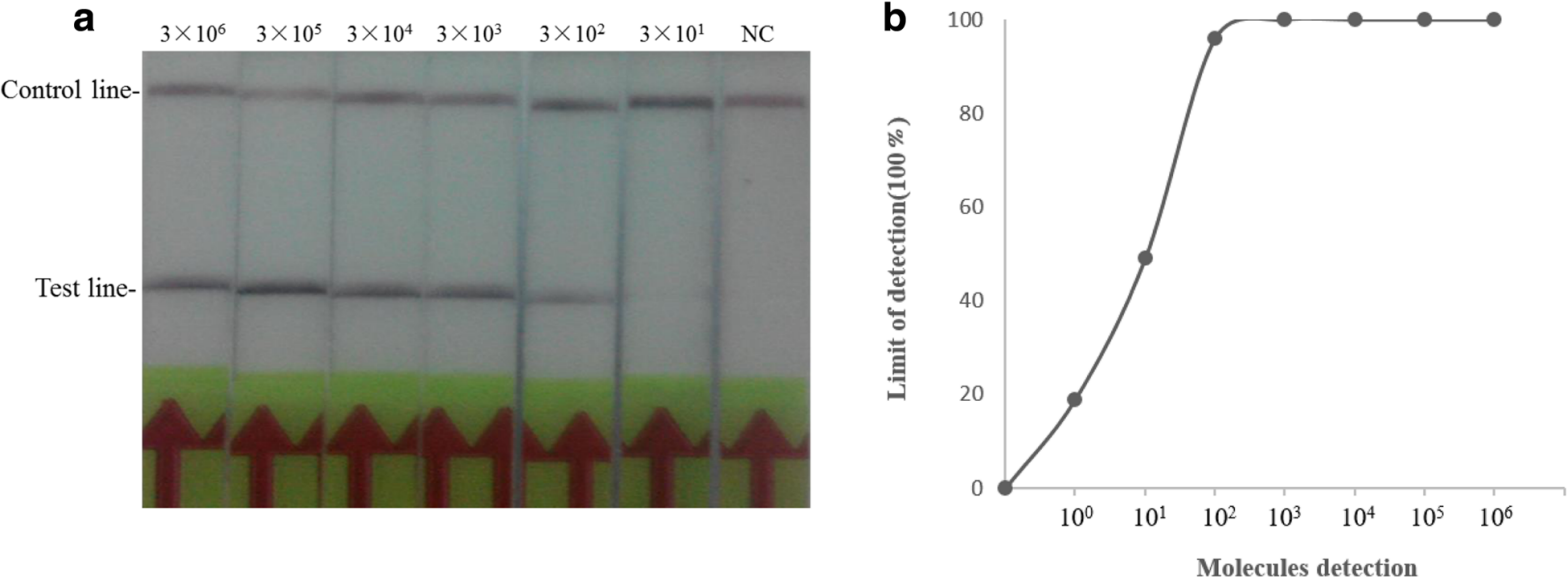
Sensitivity of CaPV RPA LFD assay (**a**) The sensitivity of CaPV RPA LFD assay was performed using a dilution series of the pCaPV/RPA DNA, and NC represents negative control; (**b**) The limit of detection in 95% probability based on eight replicates of CaPV RPA LFD assay

In exploring the optimal temperature for amplification in CaPV RPA LFD assay, it was found that the target viral DNA gene can be amplified well from 30 °C to 45 °C and could be detected in more than 10 min (Fig. 4a, b). The sensitivity of the CaPV RPA LFD assay was determined using serial dilutions of the purified reference DNA as described above. As shown in Fig. 5a, the sensitivity of CaPV RPA LFD assay was 3 × 10^2^ copies per reaction (Fig. 5a). The limit of detection in 95% probability was 3 × 10^2^ copies per reaction (probit analysis, *p* ≤ 0.05) (Fig. 5b). To further determine its sensitivity, it was evaluated using SPPV-spiked samples (*n* = 24). Positive band on the LFD was observed for all spiked samples (Additional file 1: Table S1). The detection limit of CaPV RPA LFD assay was also tested on SPPV/Gulang 2009 gDNA (genomic DNA), and the results showed that it could detect as low as 10^3^ copies per reaction (Additional file 2: Figure S1). In investigation of the specificity of CaPV RPA LFD assay, the reactions were performed using a panel of genomes extracted from other important viruses of small ruminants as described above, which caused similar clinical signs. As shown in Table 2, the CaPV RPA LFD assay was specific for the detection of GTPV and SPPV. The CaPV RPA LFD assay also performed well on DNA extracted from spiked tissues samples using a simple DNA preparation method as described above (Table 3). And there was no difference of detection sensitivity between the two extraction methods when tested by CaPV RPA LFD assay (Table 3 and Additional file 1: Table S1).

### Performance of CaPV real-time RPA and RPA LFD assay on clinical samples

All the clinical samples (*n* = 107) were detected simultaneously by CaPV real-time RPA assay, CaPV RPA LFD assay and CaPV real-time qPCR assay. Thirty-six samples were determined to be positive by CaPV real-time RPA assay (threshold time ranging from 4 to 6.6 min) and CaPV RPA LFD assay, while thirty-seven samples were positive by CaPV real-time qPCR assay (CT value ranging from 18 to 29). The clinical sensitivity and specificity of CaPV real-time RPA assay and CaPV RPA LFD assay for identification of GTPV and SPPV were 97% and 100%, respectively, when compared to CaPV real-time qPCR assay (Table 4).

**Table 4:** Comparison of CaPV real-time RPA assay and CaPV RPA LFD assay with real-time qPCR assay on clinical samples

clinical sample	Real-time RPA		RPA LFD		Real-time (qPCR)	
	Positive	Negative	Positive	Negative	Positive	Negative
Liver	6	12	6	12	6	12
Lung	5	9	5	9	5	9
Kidney	12	13	12	13	12	13
Spleen	5	5	5	5	6	4
Skin	5	15	5	15	5	15
Blood	3	17	3	17	3	17
Total	36	71	36	71	37	70

## Discussion

This study shows that the developed CaPV real-time RPA assay and CaPV RPA LFD assay are specific for detection of SPPV and GTPV with a detection limit of three hundred copies within 20 min. The sequence alignment shows these two methods would be also capable of detecting LSDV, which causes lumpy skin disease in cattle. However, their effectiveness for detecting LSDV in China remains to be determined as China is free of LSDV. RPA technology has multiple advantages over real-time qPCR assays. RPA does not require expensive equipment for amplification and has quicker time-to-answer under low temperature. These characteristics of the RPA assay make it much more applicable for field detection, in an infrastructure limited rural area, or for rapid diagnosis in less well-equipped laboratories. For the CaPV RPA LFD assay developed in this study, only a water bath is required for the RPA assay itself, and the amplicons analysis could be performed simply on the LFD strip which can be read by the naked eyes. A simple point-of-care scanner (ESEQuant tube scanner device, Germany) can also be used in the real-time RPA assay [13, 14]. The scanner is much cheaper and simpler than thermal cycler machine, and is also powered by battery which can be changed in the field.

For pen-site diagnostics, isothermal amplification technologies are of great interest due to their convenience or simplicity. Of these methods, a loop-mediated isothermal amplification (LAMP) has been studied for CaPV detection. The first CaPV LAMP assay targeted CaPV P32 gene was designed to detect both SPPV and GPPV as well as LDSV, and had a detection limit of 163 DNA copies per μL [19, 20], which is equivalent to the performance of the developed RPA assay in this study. In contrast to RPA assay, LAMP assay requires a longer time (45–60 min), a higher temperature (60–65 °C) and more complex primers (three pairs of primers) [19, 20]. Moreover, LAMP assay employs non-specific SYBR Green-based detection while RPA assay employs specific probes detection.

## Conclusions

The CaPV real-time RPA assay and CaPV RPA LFD assay are successfully developed for the rapid and specific detection of SPPV and GTPV. Both assays could specially detect SPPV or GTPV present in liver, lung, kidney, spleen, skin and blood. The results are encouraging but the assay must be validated by analysis of a larger number of samples. With further optimization and validation, the RPA has potential to be a promising alternative to real-time qPCR or other isothermal methods for rapid detection of SPPV and GPPV or could be used in the field or in an infrastructure limited rural area.

## Additional files

Additional file 1: Table S1. Comparison of CaPV real-time RPA assay and CaPV RPA LFD assay with real-time qPCR assay on spiked samples. (DOCX 18 kb)

Additional file 2: Figure S1. The detection limit of CaPV RPA LFD assay. This assay was performed using a dilution series of the SPPV/Gulang 2009 genomic DNA, and NC represents negative control. (PPTX 393 kb)

## Abbreviations

CaPV: Capripoxvirus
GTPV: Goatpox virus
Real-time qPCR: Real-time quantitative PCR
Real-time RPA assay: Recombinase polymerase amplification assay using real-time fluorescent detection
RPA LFD assay: Recombinase polymerase amplification assay combination with lateral flow dipstick
RPA: Recombinase polymerase amplification
SPPV: Sheeppox virus
