# Supplementary material for: Development of real-time and lateral flow dipstick recombinase polymerase amplification assays for rapid detection of goatpox virus and sheeppox virus
Source: Virol J. 2017 Jul 17;14:131. doi: 10.1186/s12985-017-0792-7 (PMC5514530; doi:10.1186/s12985-017-0792-7)
Supplement: Supplementary file 1 — Comparison of CaPV real-time RPA assay and CaPV RPA LFD assay with real-time qPCR assay on spiked samples. (DOCX 18 kb) [file 12985_2017_792_MOESM1_ESM.docx]

Table S1. Comparison of CaPV real-time RPA assay and CaPV RPA LFD assay with real-time qPCR assay on spiked samples

| Spiked samples name | CaPV real-time RPA(min) | CaPV RPA LFD | real-time qPCR(CT) |
| --- | --- | --- | --- |
| Liver 1 | 3.6 | + | 21 |
| Liver 2 | 7 | + | 27 |
| Liver 3 | 6.3 | + | 26 |
| Lung 1 | 7.6 | + | 31 |
| Lung 2 | 7.3 | + | 29 |
| Lung 3 | 4 | + | 24 |
| Stomach 1 | 4.3 | + | 26 |
| Stomach 2 | 6.6 | + | 28 |
| Stomach 3 | 8 | + | 30 |
| Kidney 1 | 4 | + | 22 |
| Kidney 2 | 4.6 | + | 25 |
| Kidney 3 | 7.3 | + | 29 |
| Lymphatic nodes 1 | 8 | + | 30 |
| Lymphatic nodes 2 | 3.6 | + | 23 |
| Lymphatic nodes 3 | 3.6 | + | 23 |
| Spleen 1 | 4.3 | + | 24 |
| Spleen 2 | 6 | + | 26 |
| Spleen 3 | 7.3 | + | 27 |
| Skin 1 | 4 | + | 22 |
| Skin 2 | 6.3 | + | 27 |
| Skin 3 | 6.3 | + | 28 |
| Nasal swab 1 | 5.6 | + | 25 |
| Nasal swab 2 | 6.3 | + | 26 |
| Nasal swab 3 | 7.3 | + | 28 |

+ : positive
