# Supplementary figures and images for: Development of real-time and lateral flow dipstick recombinase polymerase amplification assays for rapid detection of goatpox virus and sheeppox virus
Source: Virol J. 2017 Jul 17;14:131. doi: 10.1186/s12985-017-0792-7 (PMC5514530; doi:10.1186/s12985-017-0792-7)

## Slide 1
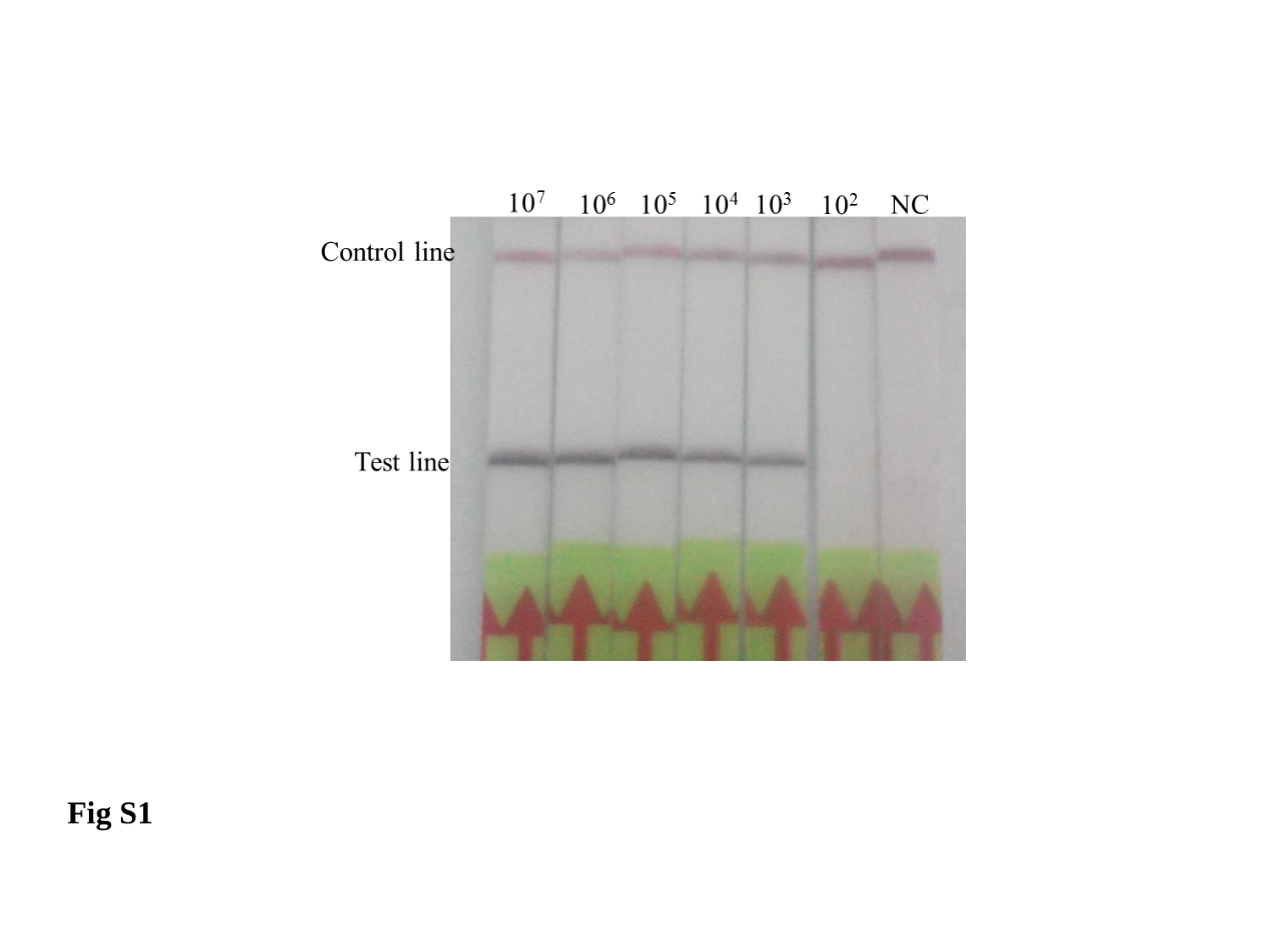

Fig S1

Supplement: Supplementary file 2 — The detection limit of CaPV RPA LFD assay. This assay was performed using a dilution series of the SPPV/Gulang 2009 genomic DNA, and NC represents negative control. (PPTX 393 kb) [file 12985_2017_792_MOESM2_ESM.pptx]
